# Engineered Nanocomposite Coatings: From Water-Soluble Polymer to Advanced Hydrophobic Performances

**DOI:** 10.3390/ma17030574

**Published:** 2024-01-25

**Authors:** Syrine Jebali, Marylène Vayer, Khaled Belal, Christophe Sinturel

**Affiliations:** 1 Interfaces, Confinement, Matériaux et Nanostructures (ICMN), CNRS-Université d’Orléans, UMR 7374, 45071 Orleans, France; syrine.jebali@polytechnique.edu (S.J.); marylene.vayer@univ-orleans.fr (M.V.); 2 Kemica Coatings, Za du Bois Gueslin, 28630 Mignieres, France; khaled.belal@kemica-coatings.com

**Keywords:** hydrophobic coating, hydrophilic polymer, polyvinylpyrrolidone, UV crosslinking, micro- and nanostructuration

## Abstract

In this work, a water-soluble (hydrophilic) polymer was used to form a hydrophobic coating on silicon substrates (Si) in a two-step process comprising (i) the transformation of the polymer into an insoluble material and (ii) the structuring of this coating at nanometric and micrometric scales to achieve the desired hydrophobic behavior. Polyvinylpyrrolidone (PVP), a water-soluble commodity polymer, was crosslinked using benzophenone and UV irradiation to produce a water-insoluble PVP coating. The nanometric scale roughness of the coating was achieved by the addition of silica nanoparticles (NPs) in the coating. The micrometric scale roughness was achieved by forming vertical pillars of PVP/NP coating. To prepare these pillars, a perforated polystyrene (PS) template was filled with a PVP/NP suspension. Micrometer scale vertical pillars of PVP/silica NPs were produced by this method, which allowed us to tune the wettability of the surface, by combining the micrometric scale roughness of the pillars to the nanometric scale roughness provided by the nanoparticles at the surface. By adjusting the various experimental parameters, a hydrophobic PVP coating was prepared with a water contact angle of 110°, resulting in an improvement of more than 80% compared to the bare flat film with an equal amount of nanoparticles. This study paves the way for the development of a more simplified experimental approach, relying on a blend of polymers containing PVP and NPs, to form the micro/nano-structured PVP pillars directly after the deposition step and the selective etching of the sacrificial major phase.

## 1. Introduction

The development of polymer coatings with water repellent properties is an active area of research today, for their anti-fouling, self-cleaning and anti-frost properties, just to name a few [1,2]. The domains of applications are very diverse and concern the automobile, aeronautic, construction, maritime, photovoltaic panel, textile industries, etc. “Biofouling” of boat hulls limits the proliferation of algae on their surface, the anti-fouling of tanks and water tanks makes them easier to clean, and the “anti-ice” effect (avoiding ice adhesion) is beneficial in the case of wind turbine blades, allowing the reduction of hydrodynamic drag. From a technical point of view, the wettability of a coating depends on the molecular interactions between the liquid and the coating surface and depends not only on the chemical nature of the substrate but also on its structuration [1].

In terms of the chemical nature, low surface energy polymers are usually sought, such as polytetrafluoroethylene [3] or polydimethylsiloxane (PDMS) [4], but this is not mandatory and a wide variety of hydrophobic polymers can also be used, including polyurethane [5], polystyrene, polyethylene or polycarbonate [4,5,6,7]. Preparing hydrophobic or superhydrophobic coatings with hydrophilic polymers (i.e., water-soluble polymers) is contradictory and is thus a real challenge. Some attempts have been made to use polyvinyl alcohol (PVA), but in this case, the polymer is fully covered or modified by a hydrophobic layer. Gong and co-workers [8] used it to immobilize hydrophilic silica nanoparticles on a substrate, with the surface being further modified by PDMS. Bai and co-workers used a PVA sponge but it was modified by stearic acid/nano-TiO_2_ particles [9].

In addition to the chemical nature of the polymer, the surface topography also has a strong impact on the wettability of the material. It is well known that surface roughness drives the wettability of a surface and that hierarchical structures that encompass both micro- and nanoscale features enable hydrophobic or superhydrophobic surfaces to be obtained. 

In general, two main approaches have been reported to structure the surface of polymers. The first approach involves modifying the polymer’s surface to increase its roughness without introducing any additional materials, while the second approach requires embedding nanoparticles in the coating.

With the first approach, polymer surface structuring has been achieved using a wide variety of methods. Some are physical ones, such as plasma treatment [3], laser irradiation [8], ionic beam irradiation [10] or polymer molding processes [4,11]. Others are more chemical, as in the following examples, where the hydrophobic behavior of the surface arises from processes leading to a pronounced increase in roughness as solvent /non-solvent casting [12,13,14] or phase separation of two immiscible polymers [15].

The second approach involves the introduction of structuring agents such as inorganic or organic particles into the polymer or onto its surface, such as the introduction of PVDF particles in a polycarbonate matrix [16] or nanotubes in epoxy resin [17]. The use of functionalized silica particles is also widespread in polymer coating [18,19,20,21,22,23] or on polymer film [24]. 

Some methods can combine the methods mentioned above. Jiang and co-workers [5] applied a “salt method” to obtain a rough PDMS by introducing NaCl particles into the PDMS before crosslinking and dissolving the NaCl after crosslinking. Other authors [25,26,27] proposed to combine the use of a solvent /non-solvent and the introduction of hydrophobic silica nanoparticles.

The challenge in the present work was to prepare a hydrophobic coating using a water-soluble hydrophilic polymer, polyvinylpyrrolidone (PVP). This approach aligns with green chemistry principles, emphasizing sustainability and environmental friendliness by utilizing water as a solvent. PVP, being a biodegradable and non-toxic polymer, emerges as an eco-friendly choice for surface modification. Although PVP is intrinsically hydrophilic, it can be modified to become hydrophobic in order to meet certain application needs. Notably, PVP is well known for its biocompatibility, making it an excellent option for pharmaceutical and biomedical applications [28,29]. Moreover, PVP exhibits exceptional film-forming capabilities [30]. When made hydrophobic, these films act as efficient moisture barriers and can be used in packaging and protective coatings. The versatility of PVP extends to its compatibility with nanoparticles [31] facilitating the development of composite materials with enhanced properties, as demonstrated in this article.

The chosen method is based on the preparation of a rough hydrophilic polymer nanocomposite coating (polymer + nanoparticles) on the top of a silicon wafer, followed by crosslinking of the polymer to render the coating insoluble in water and silanization of the nanoparticles to render the coating hydrophobic. To improve the hydrophobic properties, we prepared micrometer scale vertical pillars of PVP nanocomposites in order to add a micrometric scale roughness to the nanometric scale roughness provided by the nanoparticles at the surface. This was obtained by filling a perforated polymer template previously formed by depositing a polystyrene/polylactic acid (PS/PLA) blend, followed by selective removal of the minor component.

## 2. Materials and Methods

### 2.1. Polymer and Organic Materials

Polystyrene (PS), with a molar weight of 350 kg·mol^−1^, Ludox^®^AS-40 colloidal silica suspension (40 wt.% SiO_2_ suspension stabilized with ammonium in water), hexamethyl disilazane (HMDS), benzophenone and all the solvents were purchased from Sigma Aldrich (Saint Quentin Fallavier, France) and used as received. Polyvinylpyrrolidone (PVP), Mw = 58 kg·mol^−1^, was purchased from Thermo Fisher Gmbh Karlruhe, Germany and polylactic acid (PLA), Mw = 25 kg·mol^−1^, from Atakina Company, West Lafayette, IN, USA. Ludox AS-40 particles were characterized by transmission electron microscopy (TEM), atomic force microscopy (AFM) and dynamic light scattering (DLS). TEM and AFM images were presented in Figure S1 and DLS data in Figure S2. As in a previous paper [32], the particle size of Ludox AS-40 silica determined by TEM was 26 ± 2 nm. In suspension in ethanol, DLS data analyses led to an average diameter of NPs in ethanol of 38 ± 4 nm and in A10 suspension of 68 ± 4 nm.

### 2.2. Wafer and Substrates Preparation

Si (100) 6″ wafers were supplied by MEMC Electronic Materials, Colombes, France and cut into 10 × 70 mm^2^ substrates. All substrates were prepared from a single wafer to ensure the reproducibility of surface composition, as it is known that the nature of the oxide layer at the top of the Si substrate can strongly influence the wetting behavior and hence the morphology of heterogeneous polymer systems deposited as thin films. The substrates were first cleaned with acetone and ethanol, dried under dry nitrogen gas for 1 min and used immediately.

### 2.3. Polymer Solutions and Nanocomposite Films Preparation

- Solution of PVP and nanocomposites suspension.

To prepare the PVP polymer solution and nanocomposite suspensions, ethanol was chosen as the solvent rather than water as ethanol is more volatile and therefore leads to a better-quality film.

A PVP solution (50 mg·mL^−1^) was prepared by first mixing benzophenone and ethanol at room temperature and stirring the solution, then adding the polymer and stirring the solution again under magnetic stirring for 1 h. This solution will be referred to as A0 for the remainder of this paper.

For the nanocomposite suspension, a diluted suspension of silica nanoparticles (NPs) in water (NP 20 wt.%) was first prepared from the commercial suspension Ludox AS-40. Benzophenone was first weighed (5 wt.% of PVP) and dissolved in ethanol. A given weight of dilute silica nanoparticle suspension was then added and the resulting suspension stirred before adding a weighted amount of PVP to obtain a suspension at 50 mg·mL^−1^ of PVP in ethanol and known mass ratios between NP and PVP in the dried films of 10:100, 30:100 and 50:100 (NP/PVP mass ratio). These suspensions will henceforth be referred to as A10, A30 and A50. As an illustration, to prepare 15 mL of PVP/NP suspension (50 mg·mL^−1^) with an NP/PVP mass ratio of 10:100 (A10), 37.5 mg of benzophenone was first dissolved in 11.5 g of ethanol. Then, 375 mg of the colloidal solution of Si NP (NP 20 wt.%), thus containing 75 mg of Si NP, was dispersed in the alcoholic solution before adding 750 mg of PVP. The suspensions were then stirred for at least 1 h. Transparent suspensions were obtained, indicating the absence of sedimentation and aggregation, and were used on the same day. The compositions of the solution and suspensions prepared are shown in Table 1.

- Solution of PS and PLA blends.

PS and PLA solutions were prepared by dissolving the adequate mass of polymer in solvent (tetrahydrofuran (THF)) to obtain the adequate polymer concentration (50 mg·mL^−1^) and mass ratio (namely, PS:PLA 60:40) The solutions were stirred for at least 2 h.

- Selective removal of one polymer PLA.

PLA was removed by depositing a drop of glacial acetic acid on the film for 15 s and wiping it out with a stream of N_2_.

- Selective removal of one polymer PS.

PS was removed by depositing a drop of tetrachloroethylene on the film for 15 s and wiping it out with a stream of N_2_.

- Dip-coating film deposition.

Thin films of polymer nanocomposites were prepared at room temperature on a silicon substrate by dip-coating (dip-coater QPI-168, Qualtech Industrial Products, Manchester, UK). The same container (diameter = 2.5 mm, total height = 57 mm) and the same volume of solution (15 mL) were always used to keep constant deposition conditions.

### 2.4. Post-Treatments of the Samples

- UV curing of the film.

UV curing was carried out using a Herolab UV lamp type NU 254–365 nm, 4 W, 500 µW/cm^2^ (Herolab GmbH, Wiesloch, Germany). Exposure time was set to 15 h, which represents the minimum time required for our system to achieve a stable PVP film resistant to dissolution in water (shorter irradiation times were not sufficient to achieve the film crosslinking). This long exposure time (15 h) compared to the times given in the literature (between a few min and a few h) was due to the very low lamp power used (4 W) compared to the lamp powers (between 20 W [33] and 400 W [34]) generally used. The UV irradiation wavelength used was 365 nm, which is optimal for exciting benzophenone groups [35,36]. The samples were placed under the lamps at a distance of 2 cm.

- UV-ozone treatment.

Ozonolysis was carried out by ultraviolet-ozone (UVO) treatment (UVO Cleaner by Jelight (Jelight Company Inc., Irvine, CA, USA). Thin films were exposed to ozone for 15 min (the distance between the UV lamp and the sample was set to 5 cm) and rinsed in distilled water.

- HMDS silanization.

Silanization was carried out using HMDS. HMDS was mixed with toluene (1 part of HMDS to 5 parts of toluene) and introduced into a closed box. The sample was immersed in this solution at room temperature for 15 h and then thoroughly rinsed with clean toluene.

### 2.5. Atomic Force Microscopy

Atomic Force Microscopy (AFM) images were collected using Dimension Icon with ScanAsyst from Bruker (Palaiseau, France). AFM imaging was performed in air at room temperature in tapping mode. TESPA-300 silicon cantilevers (resonance frequency 320 kHz, spring constant 40 N·m^−1^) were used. Only height images were recorded and they were used to examine the topography of the film surface. For morphologies with circular domains, AFM images were thresholded to obtain binary images and evaluate the characteristics of the discrete domains. The surface covered by the discrete domains and the area, length and geometric centers of the discrete domains were determined using Image J software [37] and the domains were fitted by an ellipse. The size of the domains was calculated as the mean between the lengths of the major and minor axes.

The films were scratched after dip-coating in order to partially uncover the bare substrate. The scratched area was imaged by AFM and cross-sectional profiles were performed. The corresponding profiles were used to evaluate the thicknesses of the films.

### 2.6. Contact Angles Measurement

Contact angles were measured on a GBX contact angle system (GBX Scientific Ltd., Romans sur Isère, France) at room temperature. Droplets of water of 2 µL were carefully deposited on the surface of the samples and average contact angles were obtained by measuring at five different positions on the sample.

### 2.7. Scanning Electron Microscopy

Scanning Electron Microscopy (SEM) was performed on a JEOL microscope JSM 7900 F (JEOL SAS, Croissy, France), with a beam energy of 15 keV. Cross-sectional views were taken on samples fractured at room temperature (22 °C), with a tilt angle of 15°.

## 3. Results and Discussion

### 3.1. Preparation of Water-Insoluble PVP Films

For PVP thin films, a common method used is crosslinking by introducing a photoinitiator (hydrogen peroxide or benzophenone) into the film, and/or using UV exposure [38,39,40,41]. In this work, benzophenone was used as an initiator. As demonstrated in numerous papers, UV irradiation of PVP thin films induces two antagonistic phenomena: crosslinking and PVP degradation [34,40]. Benzophenone, acting as a molecule sensitive to light, undergoes excitation upon absorption of a UV photon with a wavelength ranging from 250 to 365 nm. This excitation leads to the generation of a ketyl radical, which engages with the PVP molecule [40,42]. Typically, the carbon atom adjacent to the nitrogen atom in the lactam ring and the carbon atom in the polymer chain bearing the lactam ring are the two possible locations for radical production in the PVP molecule that occurs due to the hydrogen atom abstraction [39]. Eventually, all the generated radicals recombine, forming a polymer network through various crosslinking bonds. In the presence of oxygen, the radical can also undergo oxidation [40], evolving towards chain scission and, thus, polymer degradation.

**Figure 1:**
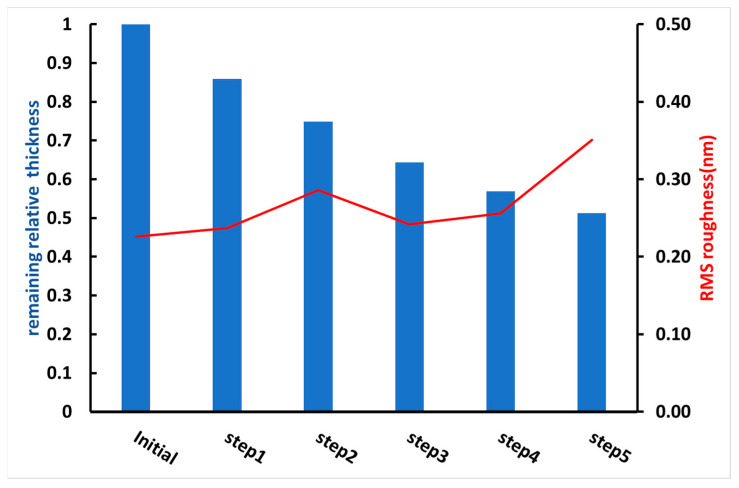
Modification of thickness and roughness of a thin film of PVP as a function of number of steps of 15 h UV irradiation, water washing and drying (initial film thickness was 200 nm). The RMS roughness was determined on a surface area of 1 × 1 µm^2^.

A compromise must be found between these two phenomena, i.e., sufficiently dense PVP crosslinking to produce insoluble thin films with minimal PVP degradation. To this end, a protocol with repetitive steps was tested, with each step consisting of a 15 h UV irradiation, water washing of the film and drying. Before irradiation and after each step, the topography and the thickness of the film were characterized by AFM, and the solubility of the PVP film in water was tested by washing with water. Figure 1 shows the evolution of thickness and roughness of PVP thin films deposited on the surface of silicon wafers, as a function of number of steps. After only one step, the film is already insoluble and the loss of thickness is 20% of the initial thickness (we checked that an irradiation time below 15 h is not sufficient to obtain a crosslinked matrix). After five steps, the film is still in place but with a 50% reduction in thickness. The best compromise is therefore one step, limiting the reduction in thickness and leading to an insoluble film. The water contact angle of these films, whatever the number of steps, ranged between 20 and 29° and still resulted in a hydrophilic but water-insoluble surface.

The first way to improve the hydrophobicity of these water-insoluble PVP crosslinked films is to introduce functionalized NPs on their surface.

### 3.2. Introduction and Functionalization of Silica NPs in the PVP Polymer

**Figure 2:**
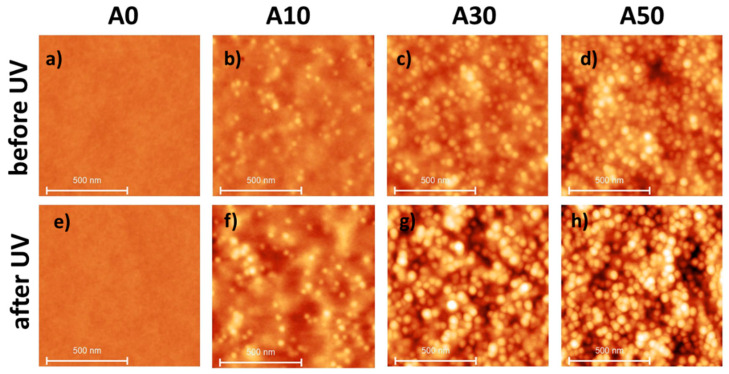
AFM images measuring 1 × 1 × µm^2^ of the thin film of neat PVP (A0) and composites (A10, A30, A50) before (**a**–**d**) and after UV irradiation (**e**–**h**). Z-scale for all images is 20 nm. Withdrawal speed for dip-coating is 50 mm·min^−1^.

An easy way to introduce nanoparticles on the film surface is to add them to the solution that is deposited by dip-coating. In a previous paper, it was shown that particles are rather well dispersed when the withdrawal speed chosen is in the draining regime, far from the critical speed between the capillary and the draining regime [32]. Up to 50% by weight of bare NPs (unfunctionalized) were introduced into the solution. Figure 2a–d shows 1 × 1 µm AFM images of the film surface after deposition. The surface of the neat PVP film remained completely smooth. In contrast, circular prominences are observed on the surface of the nanocomposite films, revealing the presence of NPs. As previously demonstrated [32], these topographic features do not correspond to naked particles emerging from the surface. They are in fact the consequence of particles buried in the near surface layer (~10 nm), inducing a surface deformation whose amplitude depends on the particle depth. Outside this layer (deeper in the film), NPs do not provoke any surface deformation and thus are not detected in AFM. As illustrated by the AFM observations, the concentration of particles in the suspension influences the number of particles on the surface. Surface thickness and roughness were measured and are presented in Table 2. The thickness before UV increased slightly with the NP concentration due to an increase in the viscosity of the suspension. The roughness increased with the weight percentage of NPs in the suspension due to a modification of the topography of the surface due to the presence of the NPs. Note that, because the films are not crosslinked before the UV irradiation, the water contact angle (WCA) cannot be measured.

In order to prepare insoluble films, the films were exposed to UV irradiation. We verified that 15 h of irradiation (optimal time for the A0 film containing only PVP) was sufficient to obtain water-insoluble composite films. As shown in Table 2 and Figures S3 and S4, UV irradiation reduces the film thickness, indicating that the superficial layer of the film is degraded. It consequently makes the silica NPs more visible (see AFM images Figure 2e–h), reflecting a higher roughness (see Table 2). The greater the concentration of nanoparticles in the suspension, the more pronounced these phenomena are. As an example, for an A50 suspension, the thickness is reduced by 40% and roughness is multiplied by 2. The WCA can be measured since the films are now insoluble in water, and it varies between 20 and 29° (see Table 2). This low WCA is consistent with the hydrophilic nature of irradiated PVP [43] and the surface chemistry of the silica NPs. It can be concluded at this stage that UV irradiation can be used to prepare water-insoluble hydrophilic PVP/NP film.

**Figure 3:**
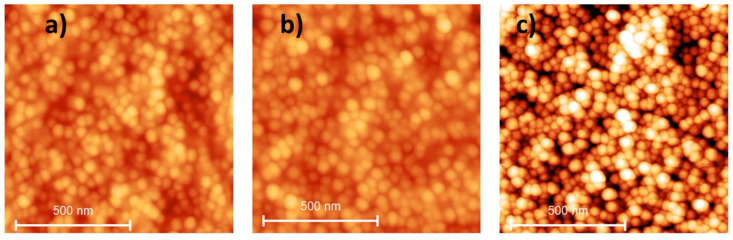
AFM images measuring 1 × 1 µm^2^ of an A50 film (**a**) after 15 h UV irradiation, (**b**) after protocol A and (**c**) after protocol B. Z scale is 50 nm.

To make the films hydrophobic, a protocol (referred to as protocol A) with UV irradiation and HMDS exposure was tested. Silanization with HMDS is a well-known procedure to confer hydrophobicity to silica particles [44,45]. Figure 3a,b present the topographical AFM images of the A50 film surface after UV irradiation and HMDS treatment (protocol A). This treatment of the film does not induce any visible modification of the surface.

HMDS exposition of the surface did not modify either the thickness or the roughness of the surface. However, the hydrophobicity of the film was slightly enhanced from 20 to 29° after UV irradiation to 34–47° after HMDS exposure (see Table 2 and Figure S3). Since the WCA of neat silica particles treated with HMDS is 105°, this limited increase in the WCA of the nanocomposite surface could be explained by the fact that silica NPs are still partially covered by a PVP polymer layer, which makes these particles unavailable for HMDS functionalization.

To uncover the surface of the particles and make them available for functionalization, a more stringent treatment (referred to as protocol B), adding an O_3_ exposure step between the UV irradiation and the HMDS functionalization of the film, was used. With this protocol, the surface of the film was strongly modified, as shown in Figure 3c, with a significant increase in the film roughness. As presented in Table 2 and Figure S3, the film thickness is reduced by a factor of 30% and the WCA is increased up to 90° for A50. These results are in favor of the elimination of more PVP layers on the top of the NPs (thanks to the ozone treatment), which are consequently more accessible to HMDS, leading to higher water contact angles. Additionally, an increase in hydroxyl groups after UV ozone treatment is presumed, contributing to enhanced surface functionalization [46,47]. The final WCA value (90°) is then the result of a hydrophobic trend resulting from both a chemical modification of the surface of the NPs by HMDS and nanostructuring of the surface. It remains, however, below the value of 118° (WCA of the neat silica NPs treated with HMDS) because of the presence of hydrophilic irradiated PVP. The highest reported water contact angles on smooth, low-energy surfaces terminated with -CF_3_ groups generally fall around 120° [48,49].

These results suggest that in order to obtain a PVP composite film with higher hydrophobicity, surface structuring on a micrometric scale must be performed so as to add a double roughness scale.

### 3.3. Micrometric Surface Structuring of PVP Composite Film

It is known that surface roughness (also called “structuration” in this manuscript) drives the wettability properties [50,51], and that liquid on a surface exhibits a wetting behavior. In typical cases, this behavior can be described using either the Wenzel model [52] or Cassie and Baxter model [53].

In Wenzel’s model, when a droplet is deposited on a rough surface, the area between the droplet and the surface is increased compared to the area in the case of a smooth surface. The roughness in this case increases the nature of the surface, either hydrophobic or hydrophilic (a hydrophilic surface becomes more hydrophilic and a hydrophobic surface becomes more hydrophobic). Conversely, in the Cassie and Baxter model, the wetting on a rough surface is the result of the combined influence of solid/liquid interactions and air/liquid interactions and the contact angle is the weighted result of the contact angles on both the solid and air interfaces. The contact angle increases as the air interface fraction at the interface rises. When the air interface fraction approaches 1, the system starts to closely resemble a liquid suspended in air. It is easy to understand that maintaining the Cassie–Baxter state, rather than shifting to the Wenzel state, depends on the air interface fraction [50,54]. In these cases, elevating the surface roughness leads to an improvement in the resulting water contact angle (WCA) for specific surfaces with values surpassing 90°.

The Wenzel and Cassie–Baxter regimes are rarely encountered in practical situations due to the presence of surface defects in most textures. Therefore, it is relevant to also consider a mixed wetting regime, which corresponds to a combination of both the Wenzel and Cassie–Baxter regimes. In this case, the liquid remains attached to the surface due to local wetting in a Wenzel regime.

To enhance hydrophobic properties, surfaces are designed using insights derived from the Cassie–Baxter and Wenzel models and in imitation of nature’s unique surfaces, such as lotus leaves, wings of butterflies and various other insects [50,54]. A notable feature of these surfaces, as documented in previous research, is the presence of hierarchical structures that encompass both micro- and nanoscale features [55]. These nanoscale characteristics are renowned for their important role in conferring hydrophobic or superhydrophobic properties.

Similar surface structures have been successfully obtained by several authors, underlining the important role of hierarchical structures at the micro and nano scales [18,24,56]. In most cases, no specific arrangement of the structures on the surface was achieved. For instance, a hierarchical structure was obtained through successive spin-coatings of a hydrophobic fumed silica dispersion in an organic solvent onto either a silicone–urea copolymer or a poly(methyl methacrylate) (PMMA) film [24]. This structure comprises agglomerated silica particles with a diameter of approximately 5 μm. The surface exhibits nanometer-sized roughness attributed to the individual fumed silica particles, which have dimensions in the range of 5 to 30 nm. Additionally, AFM images have also confirmed the presence of robust silica particles with heights ranging from 0.5 to 2.2 μm.

In our research, we also tried to mimic these complex designs by structuring our water-soluble polymer to feature micrometric pillars with heights measuring several hundred nanometers.

As is often proposed in the literature, efficient microstructuring requires typical pillars with micrometric or sub-micrometric diameter, center-to-center distance and height [57].

To achieve the desired micrometric structuration, we prepared micrometric pillars of PVP loaded with silica NPs. The aim was to achieve a dual structuration of the surface (at the micro and nano scales) by combining the nanostructuring of the PVP and the microstructuring of the pillars in order to attain higher hydrophobic properties. For this purpose, a micrometric template (a sacrificial perforated polymer layer) was first prepared using the polymer blend approach (see below), and then filled with a suspension of PVP and silica NPs.

**Figure 4:**
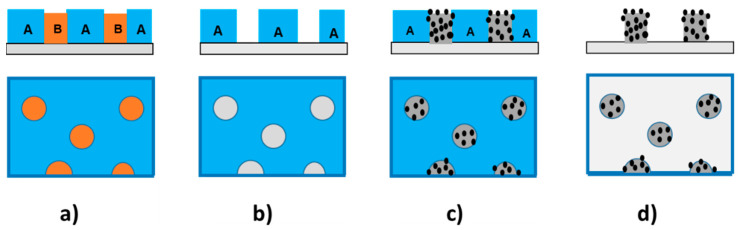
The procedure in four steps to obtain micrometric pillars. (top: sectional view, bottom: upper view). PS is blue (A), PLA is orange (B), PVP is dark grey, NPs are black and silicon substrate is light grey. (**a**) PS/PLA film exhibiting lateral phase separation (**b**) PS template after selective extraction of PLA (**c**) PS template filled with PVP/NPs nanocomposite (**d**) pillars of PVP/NP nanocomposite after extraction of the PS matrix.

Figure 4 illustrates the method used for the preparation of the PVP pillars, using the polymer blend approach. Polymer blends (A/B), when deposited as a thin film, can exhibit a well-defined and micrometric lateral phase separation under particular conditions, i.e., a matrix of one polymer A (usually the majority polymer) and cylindrical domains of the other polymer B (the minority polymer) perpendicular to the surface of the substrate (Figure 4a). If one polymer can be extracted without removing the other, then a micrometric polymer template can be prepared. If component B is extracted, a matrix of A with holes in place of the domains of B is obtained (Figure 4b). Filling the latter template with a polymer composite material (Figure 4c) (in our case, an NP/PVP composite suspension) and selective extraction of polymer A leads to micrometric polymer composite pillars in place of the holes (Figure 4d).

The blend of PS and PLA is a polymer blend which, after deposition of a THF solution of PS and PLA by dip-coating, exhibits lateral phase separation [58,59]. As PLA and PS can be easily and selectively removed by acetic acid and tetrachloroethylene, respectively, they were selected to produce polymer templates. THF was chosen as a common solvent of PS and PLA because it is a good solvent for both polymers and has a sufficiently high vapor pressure to ensure lateral rather than vertical phase separation [58,59].

The proportion between PS and PLA (PS:PLA) should be chosen to obtain a matrix of PS with discrete domains of PLA (i.e., PLA is the minority component) in order to obtain a perforated layer of PS after selective etching of the PLA. The reverse (PS as the minority component) is not appropriate because this would result in a perforated layer of PLA (after selective etching of the PS) that would require the use of acetic acid used in step (d) of Figure 4, which could damage the PVP nanocomposite (PVP is miscible in acetic acid). Moreover, the phase-separated film should exhibit micrometric domains with a sufficiently high surface coverage, preventing the use of a very low PLA concentration. For that purpose, PS-PLA films were prepared using a solution of PS:PLA (60:40) with 50 mg·mL^−1^ in THF.

**Figure 5:**
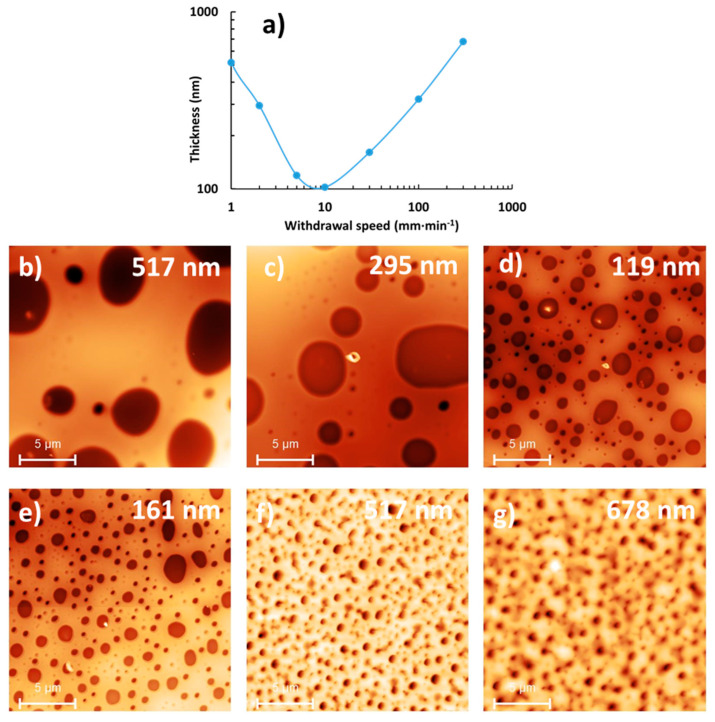
(**a**) Thickness and 20 × 20 µm^2^ topographical AFM images at a concentration of 50 mg·mL^−1^ for a PS:PLA 70:30 proportion for different withdrawal speeds: (**b**) 1, (**c**) 2, (**d**) 5, (**e**) 30, (**f**) 100 and (**g**) 300 mm·min^−1^. The thickness of the film is indicated in the top right corner of the images.

Figure 5 presents the evolution of the thickness and surface morphology of the films as a function of withdrawal speed, with the speed varying between 1 and 300 mm·min^−1^. With the withdrawal speed increase, the thickness first decreases, goes through a minimum for a critical speed and then increases again. This minimum delimits the two regimes classically observed in the dip-coating process: the capillary regime below the critical speed and the draining regime above. For the capillary regime, the AFM images display well-defined micrometric domains because the time scale is such that the system can evolve to a better-defined phase separation (low evaporation rate). In contrast, the draining regime leads to ill-defined and smaller domains, due to the time scale of the process: the system is rapidly quenched, preventing the phase separation from reaching well-defined domains. These results led us to the conclusion that the deposition of PS/PLA film must be carried out in the capillary regime: this will lead to the most efficient and well-defined phase separation without the need for an additional post-deposition step such as thermal annealing or solvent vapor annealing after dip-coating.

Upon comparing deposition speeds of 5 mm·min^−1^ and 1 mm·min^−1^, it became evident that the latter resulted in a greater film thickness. As previously discussed, high withdrawal speeds often lead to the formation of smaller and less well-defined domains, characterized by dual average sizes (Figure 5). Consequently, the deliberate choice of a 1 mm·min^−1^ deposition speed was made, as it consistently produced films with the most appropriate thickness and clearly defined micrometric domains. In these conditions, the thickness of the films is 517 ± 86 nm, the domain diameter is 5.9 ± 1.1 µm and the % hole coverage is 30.4 ± 1.4.

Once the polymer template (steps (a) and (b) of Figure 4) had been prepared, the next step of the procedure, that is filling the perforated PS template with the ethanolic suspension of PVP and NPs (step (c) of Figure 4), was performed. To this end, different concentrations of PVP:NP suspension in ethanol were tested. A concentration of 30 mg·mL^−1^ failed to fill the holes sufficiently. In contrast, a concentration of 80 mg·mL^−1^ led to a continuous coating layer on the top of the PS template. We found that an intermediate solution of 50 mg·mL^−1^ filled the holes without any top layer.

**Figure 6:**
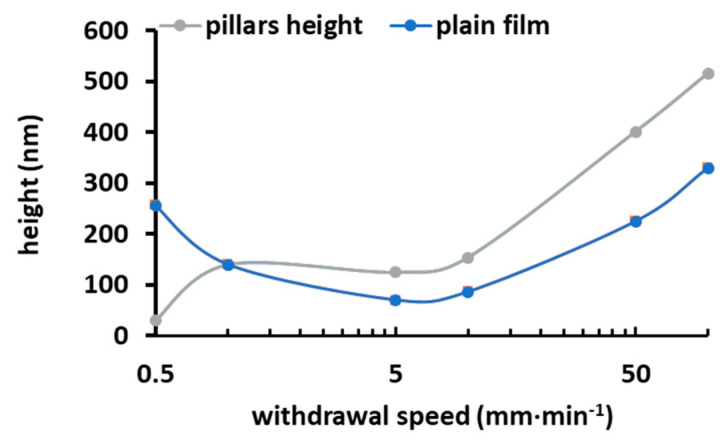
Thickness as a function of the withdrawal speed when filling the holes of the PS template and depositing plain film with A10 suspension.

Withdrawal speeds ranging from 0.5 to 100 mm·min^−1^ were tested to determine the best conditions for filling the perforated PS templates (thickness 517 ± 86 nm, diameter of the domains 5.9 ± 1.1 µm). Figure 6 displays the filling thickness (measured by the pillars’ height remaining after the etching of the template) as a function of the withdrawal speed. For comparison, the thickness of a plain thin film at different withdrawal speeds is reported, exhibiting a typical V-shaped curve.

Withdrawing at a low speed does not allow the holes of the template to be filled with the PVP/NP suspension. Since the process involves the filling of a hydrophobic porous PS template with a hydrophilic PVP/NP suspension in ethanol, this phenomenon could be explained by the fact that, in the capillary regime, the solution did not wet the template perfectly and/or was not trapped in the hole and had sufficient time to return in the suspension. Withdrawing at a high speed under the draining regime was necessary to fill the holes (more than 50 mm·min^−1^). At this withdrawal speed, the solution trapped by the template is quickly dried and is retained by the template.

In the last step, the PS template was removed (step (d) of Figure 4) to form the PVP/NP pillars array, which was subsequently subjected to silanization functionalization through protocol A or B. The final height of the pillars was above 400 nm, with an RMS surface roughness of 120 ± 5 nm.

**Figure 7:**
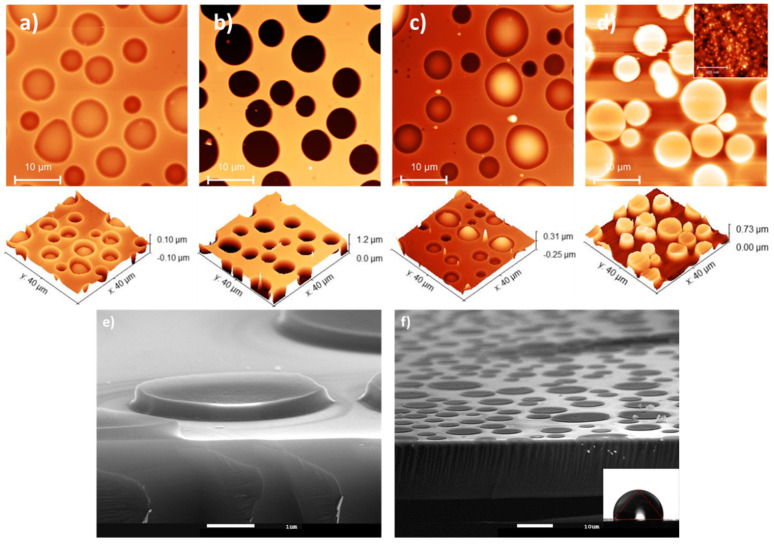
AFM and SEM images of the views of the different steps of the preparation process of a micro-/nano-structured surface: (**a**) PS:PLA thin films with lateral phase separation; (**b**) perforated PS template after selective etching of the PLA domains; (**c**) filled template with PVP:NP by dip-coating; (**d**–**f**) PVP:NP pillars obtained by selective removal of the PS template. The process conditions were the following: PS:PLA 60:40 50 mg·mL^−1^ in THF 1 mm·min^−1^, filling with A10 composite suspension in ethanol 50 mg·mL^−1^ by dip-coating at 50·mm·min^−1^.

Figure 7a–d presents the AFM images taken at different steps of the process to prepare structured surfaces of PVP at micrometric and nanometric scales with the optimal parameters. The insert in Figure 7d presents an AFM image demonstrating that NPs can be observed on the top of the pillars. Figure 7e,f present SEM cross-sectional images of the pillars obtained.

The WCAs were then measured for the A0, A10 and A50 suspensions and are presented in Table 3. It is worth noting here that the droplet size (approximately 1 mm) is significantly larger than the size of the pillars, covering numerous pillars in this case. The results were compared with those of the plain PVP/NP film. For all conditions, the presence of pillars induces higher WCA compared to the bare flat film with equal amounts of NPs. Even in the absence of NP (A0), it is interesting to note that the PVP pillars increase the hydrophobic properties, indicating that the microstructuring of the surface has already had an impact. The highest improvement was obtained with 10% of NPs, where the WCA jumped from 60° on a flat film to 110° on the micro-/nano-structured version, in the case of protocol B. It is worth noting that the WCA in this condition is significantly higher than that of a bare Si wafer (95°), confirming that the presence of the micro-/nano-structured pillars induces a beneficial effect on the hydrophobic properties of the substrate. Half of NPs do not show any additional effect, indicating that a concentration of 10% is sufficient to provoke the desired effect. To understand this phenomenon, the roughness on the top of the pillars was determined and was found to be 0.5, 1.0 and 1.7 nm for A0, A10 and A50, respectively. This increase is less dependent on the NP amount than for the plain films (see Table 1). This could explain why pillars filled with A10 and A50 led to similar water contact angles. This difference of behavior could be attributed to the mode of preparation of the pillars that included the filling of the pores instead of a deposition on a flat surface.

Although the mechanical behavior of the pillars was not explored in detail in this paper, we verified that the surface nanopatterning was stable upon repeated immersion and drying cycles, owing to its crosslinked structure. In addition to the loss of water solubility, it is known from the literature that PVP mechanical properties are improved with crosslinking [60]. It is also worth mentioning that composite structures bring mechanical durability due to the presence of nanoparticles, as demonstrated by Verho et al. [61].

## 4. Conclusions

In this work, we obtained a highly hydrophobic coating from a water-soluble commodity polymer (PVP). For this purpose, we prepared micrometer scale vertical pillars of PVP/silica NPs in order to combine a micrometric scale roughness with the nanometric scale roughness provided by the nanoparticles at the surface. We developed a method based on filling a perforated PS template with a PVP/NP suspension and crosslinking the PVP using benzophenone and UV irradiation to produce a hydrophobic water-insoluble PVP film. The PS template was produced by dip-coating a solution of PS/PLA in THF on a substrate. The phase separation that occurred when the substrate was withdrawn from the solution enabled a lateral phase separated matrix of PS, with cylindrical domains of PLA to be obtained directly without any further post-treatment. By adjusting the various experimental parameters, a WCA up to 110° was observed, resulting in an improvement of more than 80% compared to the bare flat film with an equal amount of NPs. Now that the proof of concept has been demonstrated, we envision simplifying the experimental approach by using a blend of polymers in water containing PVP and NPs in order to reach the micro-/nano-structured PVP pillars directly after the deposition step and the selective etching of the major phase. It is worth mentioning that this one-step procedure (all in water) could be adapted to any materials where the surface energy is high enough to be wetted by the water solution polymers/NPs (such as glass or metal). By further enhancing the roughness of the film, a prospective route emerges for attaining superhydrophobic properties. The transition from hydrophobicity to superhydrophobicity constitutes a compelling pathway for upcoming studies, paving the way towards facile realization of coating with advanced properties such as anti-fouling, self-cleaning or anti-frost.

## Figures and Tables

**Table 1:** Characteristics of the prepared solution A0 and suspensions A10, A30 and A50.

	A0	A10	A30	A50
Nominal NP:PVP wt. proportion in suspension	0:100	10:100	30:100	50:100
% vol. of PVP + NP (mL·mL^−1^) in suspension	3.83	3.82	3.78	3.74
Conc. of PVP + NP (g·mL^−1^) in suspension	0.046	0.046	0.048	0.050
% vol. of NP (mL·mL^−1^) in the dry film	0	5.4	15.2	20.0

**Table 2:** Characteristics of the films after the different treatments.

		A0	A10	A30	A50
After deposition	Thickness (nm)	100 ± 5	100 ± 5	105 ± 5	125 ± 5
	Roughness Rq ^1^ (nm)	0.2 ± 0.1	1.2 ± 0.2	2.0 ± 0.2	2.2 ± 0.2
UV irradiation	Thickness (nm)	91 ± 5	90 ± 5	90 ± 5	76 ± 5
	Roughness Rq ^1^ (nm)	0.2 ± 0.1	1.3 ± 0.2	2.8 ± 0.2	4.1 ± 0.2
	WCA (°) ^2,3^	27 ± 4	29 ± 4	20 ± 4	27 ± 3
Protocol A ^4^	WCA (°) ^2,3^	34 ± 3	41 ± 3	43 ± 3	47 ± 2
Protocol B	Thickness (nm)	79 ± 5	79 ± 5	68 ± 5	65 ± 5
	Roughness Rq ^1^ (nm)	0.2 ± 0.1	1.4 ± 0.2	6.4 ± 0.2	8.7 ± 0.2
	WCA (°) ^2,3^	35 ± 4	60 ± 4	80 ± 4	90 ± 4

^1^ Rq: root-mean square. The roughness was determined on a surface area of 1 × 1 µm^2^. ^2^ WCA: water contact angle. ^3^ As references, WCA for neat Si wafer is 25° and after protocol A and B it is 95°. WCA for neat Si NPs is 7° and after protocol A and B it is 118° (the NPs were deposited by dip-coating in ethanol in a thick layer). Measurements of WCA after film deposition without UV treatment were not possible due to PVP water solubility. ^4^ For protocol A, the thickness and roughness are not given since the values are identical to those obtained with UV irradiation.

**Table 3:** Water contact angles (°) of the pillars coating after Treatment A: after UV exposition and HMDS silanization, and after Treatment B: after UV irradiation, O_3_ exposure and HMDS functionalization. The values for the plain film without micrometric structuring are given in brackets.

	A0/Pillars	A10/Pillars	A50/Pillars
Treatment A	81 (34)	96 (41)	93 (54)
Treatment B	80 (34)	110 (60)	110 (90)

## Data Availability

Data are contained within the article and supplementary materials.

## Supplementary Materials

The following supporting information can be downloaded at: https://www.mdpi.com/article/10.3390/ma17030574/s1.

## References

[1] Zeng Q., Zhou H., Huang J., Guo Z. (2021). Review on the recent development of durable superhydrophobic materials for practical applications. Nanoscale.

[2] Ahmad D., van den Boogaert I., Miller J., Presswell R., Jouhara H. (2018). Hydrophilic and hydrophobic materials and their applications. Energy Sources A Recovery Util. Environ. Eff..

[3] Alawajji R.A., Kannarpady G.K., Biris A.S. (2018). Fabrication of transparent superhydrophobic polytetrafluoroethylene coating. Appl. Surf. Sci..

[4] Maghsoudi K., Vazirinasab E., Momen G. (2020). Advances in the fabrication of superhydrophobic polymeric surfaces by polymer molding processes. Ind. Eng. Chem. Res..

[5] Jiang C., Zhang Y., Wang Q., Wang T. (2013). Superhydrophobic polyurethane and silica nanoparticles coating with high trans-parency and fluorescence. J. Appl. Polym. Sci..

[6] Wen G., Guo Z., Liu W. (2017). Biomimetic polymeric superhydrophobic surfaces and nanostructures: From fabrication to applications. Nanoscale.

[7] Yilbas S., Khaled M., Abu-Dheir N., Al-Aqeeli N., Said S.A.M., Ahmed A.O.M., Varanasi K.K., Toumi Y.K. (2014). Wetting and other physical characteristics of polycarbonate surface textured using laser ablation. Appl. Surf. Sci..

[8] Gong X., Zhang L., He S., Jiang S., Wang W., Wu Y. (2020). Rewritable superhydrophobic coatings fabricated using water-soluble polyvinyl alcohol. Mater. Des..

[9] Bai C., Hu C., Ni P., Zhang X., Zhang W., Zhang S., Tang J., Li T., Li Y. (2023). Fabrication of robust, superhydropho-bic-superoleophilic PVA sponge by one-pot hydrothermal method for oil-water separation. Surf. Interfaces.

[10] Oh J.-H., Ko T.-J., Moon M.-W., Park C. (2014). Hee Nanostructured superhydrophobic silk fabric fabricated using the ion beam method. RSC Adv..

[11] Adithyavairavan M., Subbiah S. (2011). A morphological study on direct polymer cast micro-textured hydrophobic surfaces. Surf. Coat. Technol..

[12] Erbil H.Y., Demirel A.L., Avci Y., Mert O. (2003). Transformation of a simple plastic into a superhydrophobic surface. Science.

[13] Li X., Chen G., Ma Y., Feng Y., Zhao H., Jiang L., Wang F. (2006). Preparation of a super-hydrophobic poly(vinyl chloride) sur-face via solvent-nonsolvent coating. Polym. J..

[14] Zhang R., Wei J., Tian N., Liang W., Zhang J. (2022). Facile preparation of robust superamphiphobic coatings on complex substrates via nonsolvent-induced phase separation. ACS Appl. Mater. Interfaces.

[15] Rioboo R., Demnati I., Ali M.A., Sevkan R., De Coninck J. (2020). Superhydrophobicity of composite surfaces created from polymer blends. J. Colloid Interface Sci..

[16] Bormashenko E., Stein T., Whyman G., Bormashenko Y., Pogreb R. (2006). Wetting Properties of the Multiscaled Nanostructured Polymer and Metallic Superhydrophobic Surfaces. Langmuir.

[17] Jung Y.C., Bhushan B. (2009). Mechanically Durable Carbon Nanotube–Composite Hierarchical Structures with Superhydrophobicity, Self-Cleaning, and Low-Drag. ACS Nano.

[18] Yilgor I., Bilgin S., Isik M., Yilgor E. (2012). Tunable wetting of polymer surfaces. Langmuir.

[19] Yilgor I., Bilgin S., Isik M., Yilgor E. (2012). Facile preparation of superhydrophobic polymer surfaces. Polymer.

[20] Su H., Yang J. (2023). Design and preparation of multiple silica particles and study on superhydrophobic modified epoxy resin. J. Polym. Res..

[21] Yousefi E., Ghadimi M.R., Amirpoor S., Dolati A. (2018). Preparation of new superhydrophobic and highly oleophobic polyure-thane coating with enhanced mechanical durability. Appl. Surf. Sci..

[22] Sunny S., Vogel N., Howell C., Vu T.L., Aizenberg J. (2014). Lubricant-Infused Nanoparticulate Coatings Assembled by Lay-er-by-Layer Deposition. Adv. Funct. Mater..

[23] Ghamarpoor R., Jamshidi M. (2022). Preparation of Superhydrophobic/Superoleophilic nitrile rubber (NBR) nanocomposites contained silanized nano silica for efficient oil/water separation. Sep. Purif. Technol..

[24] Söz C.K., Yilgör E., Yilgör I. (2015). Influence of the average surface roughness on the formation of superhydrophobic polymer surfaces through spin-coating with hydrophobic fumed silica. Polymer.

[25] Seyfi J., Hejazi I., Jafari S.H., Khonakdar H.A., Simon F. (2016). Enhanced hydrophobicity of polyurethane via non-solvent in-duced surface aggregation of silica nanoparticles. J. Colloid Interface Sci..

[26] Khakbaz M., Hejazi I., Seyfi J., Jafari S.-H., Khonakdar H.A., Davachi S.M. (2015). A novel method to control hydrolytic degra-dation of nanocomposite biocompatible materials via imparting superhydrophobicity. Appl. Surf. Sci..

[27] Zhu Y., Pei L., Ambreen J., He C., Ngai T. (2020). Facile Preparation of a Fluorine-Free, Robust, Superhydrophobic Coating through Dip Coating Combined with Non-Solvent Induced Phase Separation (Dip-Coating-NIPS) Method. Macromol. Chem. Phys..

[28] Ghutepatil P., Khot V.M., Salunkhe A.B. (2022). Design of monodispersed PVP functionalized biocompatible manganese ferrite nanoparticles for hyperthermia application. Mater. Today Proc..

[29] Kurakula M., Rao G.K. (2020). Moving polyvinyl pyrrolidone electrospun nanofibers and bioprinted scaffolds toward multidisciplinary biomedical applications. Eur. Polym. J..

[30] Harinkhere D., Karma N., Choudhary K.K., Kaurav N. (2022). Structural and optical properties of PPO/PVP blended polymer film. Mater. Today Proc..

[31] Kamarudin D., Hashim N.A., Ong B.H., Che Hassan C.R., Abdul Manaf N. (2022). Synthesis of silver nanoparticles stabilised by pvp for polymeric membrane application: A comparative study. Mater. Technol..

[32] Jebali S., Vayer M., Belal K., Mahut F., Sinturel C. (2024). Dip coating deposition of nanocomposite thin films based on water-soluble polymer and silica nanoparticles. Colloids Surf. A Physicochem. Eng. Asp..

[33] Fechine G.J.M., Barros J.A.G., Catalani L.H. (2004). Poly(N-vinyl-2-pyrrolidone) hydrogel production by ultraviolet radiation: New methodologies to accelerate crosslinking. Polymer.

[34] Tajik F., Eslahi N., Rashidi A., Rad M.M. (2021). Hybrid antibacterial hydrogels based on PVP and keratin incorporated with lavender extract. J. Polym. Res..

[35] Riga E.K., Saar J.S., Erath R., Hechenbichler M., Lienkamp K. (2017). On the Limits of Benzophenone as Cross-Linker for Sur-face-Attached Polymer Hydrogels. Polymer.

[36] Christensen S.K., Chiappelli M.C., Hayward R.C. (2012). Gelation of Copolymers with Pendent Benzophenone Pho-to-Cross-Linkers. Macromolecules.

[37] Rasband W.S. ImageJ U.S. National Institutes of Health, Bethesda, Maryland, USA. 1997–2018. https://imagej.nih.gov/ij/.

[38] Grube S., Siegmann K., Hirayama M. (2015). A moisture-absorbing and abrasion-resistant transparent coating on polystyrene. J. Coat. Technol. Res..

[39] Mirek A., Grzeczkowicz M., Belaid H., Bartkowiak A., Barranger F., Abid M., Wasyłeczko M., Pogorielov M., Bechelany M., Lewińska D. (2023). Electrospun UV-cross-linked polyvinylpyrrolidone fibers modified with polycaprolactone/polyethersulfone microspheres for drug delivery. Biomater. Adv..

[40] Zhu X., Chen P.L.W., Dong J. (2010). Studies of UV crosslinked poly(N-vinylpyrrolidone) hydrogels by FTIR, Raman and solid state NMR spectroscopies. Polymer.

[41] Zhang Q.G., Hu W.W., Zhu A.M., Liu Q.L. (2013). UV-crosslinked chitosan /polyvinylpyrrolidone blended membranes for pervaporation. RSC Adv..

[42] Maciejewska B.M., Wychowaniec J.K., Woźniak-Budych M., Popenda Ł., Warowicka A., Golba K., Litowczenko J., Fojud Z., Wereszczyńska B., Jurga S. (2019). UV cross-linked polyvinylpyrrolidone electrospun fibres as antibacterial surfaces. Sci. Technol. Adv. Mater..

[43] Sun M., Qiu H., Su C., Shi X., Wang Z., Ye Y., Zhu Y. (2019). Solvent-Free Graft-From Polymerization of Polyvinylpyrrolidone Imparting Ultralow Bacterial Fouling and Improved Biocompatibility. ACS Appl. Bio. Mater..

[44] Slavov S.V., Sanger A.R., Chuang K.T. (2000). Mechanism of Silation of Silica with Hexamethyldisilazane. J. Phys. Chem. B.

[45] Rao A.V., Pajonk G.M., Bhagat S.D., Barboux P. (2004). Comparative studies on the surface chemical modification of silica aero-gels based on various organosilane compounds of the type RnSiX4-n. J. Non-Cryst. Solids.

[46] Qin W., Vautard F., Askeland P., Yu J., Drzal L.T. (2017). Incorporation of Silicon Dioxide Nanoparticles at the Carbon Fi-ber-Epoxy Matrix Interphase and Its Effect on Composite Mechanical Properties. Polym. Compos..

[47] O’Mahony T.F., Morris M.A. (2021). Hydroxylation methods for mesoporous silica and their impact on surface functionalisation. Micropor. Mesopor. Mat..

[48] Nishino T., Meguro M., Nakamae K., Matsushita M., Ueda Y. (1999). The Lowest Surface Free Energy Based on -CF3 Alignment. Langmuir.

[49] Tutejaa A., Choib W., Mabryc J.M., McKinley G.H., Cohena R.E. (2008). Robust omniphobic surfaces. Proc. Natl. Acad. Sci. USA.

[50] Murakami D., Jinnai H., Takahara A. (2014). Wetting Transition from the Cassie−Baxter State to the Wenzel State on Textured Polymer Surfaces. Langmuir.

[51] Roach P., Shirtcliffe N.J., Newton M.I. (2008). Progess in superhydrophobic surface development. Soft Matter.

[52] Wenzel R.N. (1936). Resistance of solid surfaces to wetting by water. Ind. Eng. Chem..

[53] Cassie A.B.D., Baxter S. (1944). Wettability of Porous Surfaces. Trans. Faraday Soc..

[54] Moulinet S., Bartolo D. (2007). Life and death of a fakir droplet: Impalement transitions on superhydrophobic surfaces. Eur. Phys. J. E.

[55] Elbourne A., Crawford R.J., Ivanova E.P. (2017). Nano-structured antimicrobial surfaces: From nature to synthetic analogues. J. Colloid Interface Sci..

[56] Söz C.K., Yilgör E., Yilgör I. (2015). Influence of the coating method on the formation of superhydrophobic silicone–urea surfaces modified with fumed silica nanoparticles. Prog. Org. Coat..

[57] Zheng Q., Lü C. (2014). Size effects of surface roughness to superhydrophobicity. Procedia IUTAM.

[58] Vital A., Vayer M., Tillocher T., Dussart R., Boufnichel M., Sinturel C. (2017). Morphology control in PS/PLA blends films by dip-coating deposition. Appl. Surf. Sci..

[59] Vital A., Vayer M., Sinturel C., Tillocher T., Lefaucheux P., Dussart R. (2015). Polymer masks for structured surface and plasma etching. Appl. Surf. Sci..

[60] Guo H., Yang J., Zhao W., Xu T., Lin C., Zhang J., Zhang L. (2019). Direct formation of amphiphilic crosslinked networks based on PVP as a marine anti-biofouling coating. Chem. Eng. J..

[61] Verho T., Bower C., Andrew P., Franssila S., Ikkala O., Ras R.H.A. (2011). Mechanically Durable Superhydrophobic Surfaces. Adv. Mater..

