# Supplementary material for: Engineered Nanocomposite Coatings: From Water-Soluble Polymer to Advanced Hydrophobic Performances
Source: Materials (Basel). 2024 Jan 25;17(3):574. doi: 10.3390/ma17030574 (PMC10856293; doi:10.3390/ma17030574)
Supplement: Supplementary file 1 [file materials-17-00574-s001.zip › materials-2793476-supplementary.pdf]

Engineered nanocomposite coatings: From water-soluble polymer to advanced hydrophobic performances

Syrine Jebali[1], Marylène Vayer[1], Khaled Belal[2], Christophe Sinturel[1*]

1. Interfaces, Confinement, Matériaux et Nanostructures (ICMN), CNRS-Université d'Orléans, UMR 7374, 1b, Rue de la Férollerie, C.S. 40059, 45071 ORLEANS Cedex 2, France
2. Kemica Coatings, Za du Bois Gueslin 28630 MIGNIERES, France

* Correspondence: Christophe.sinturel@univ-orleans.fr

Supplementary information (SI):

All materials and methos are described in the main text except:

Transmission Electron Microscopy
Transmission Electron Microscopy (TEM) was used to visualize the NPs shape and size dispersed in water, ethanol and after adding the polymer. A drop of each suspension was deposited on a copper TEM grid and left to dry. TEM characterizations were carried out using a Philips CM20 instrument operated at an accelerating voltage of 200 kV.

Dynamic light scattering
Dynamic light scattering (DLS) measurements were performed on the suspension with a Zetasizer Nano ZS90 (Malvern Panalytical, Palaiseau, France) on the Ludox suspension in ethanol and also on the A10 suspension used for the dip-coating experiment. The suspension was diluted with deionized water or ethanol to adjust the light strength to the measurement condition.

S1: AFM and TEM images of the Ludox AS-40

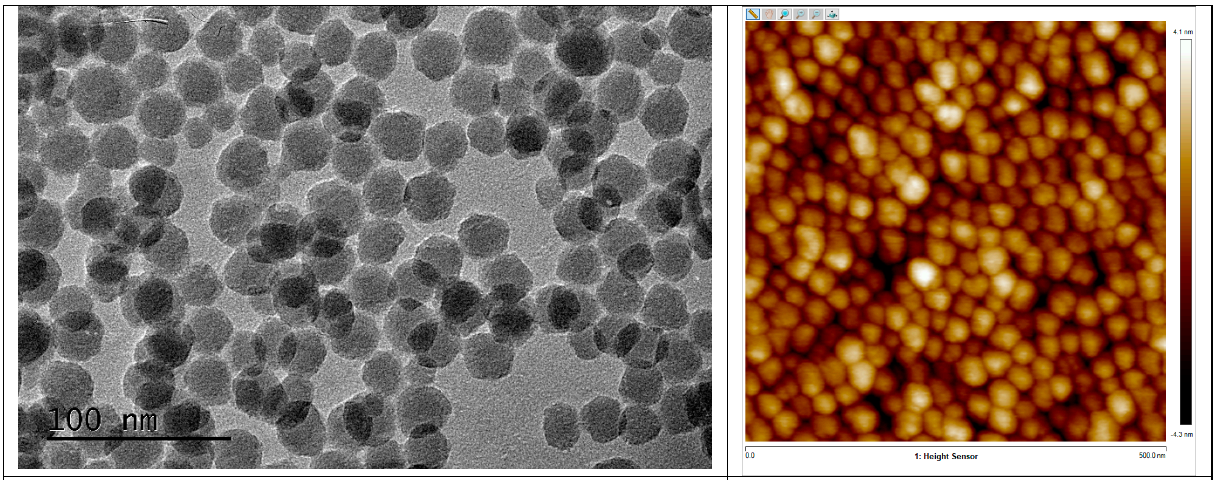

(a) (b)

Figure S1: TEM (a) and AFM (b) images of Ludox AS-40 in suspension in ethanol
In TEM images, the Ludox particles size is 26±2 nm

S2: DLS data of NPs suspension, in ethanol and in A10 suspension

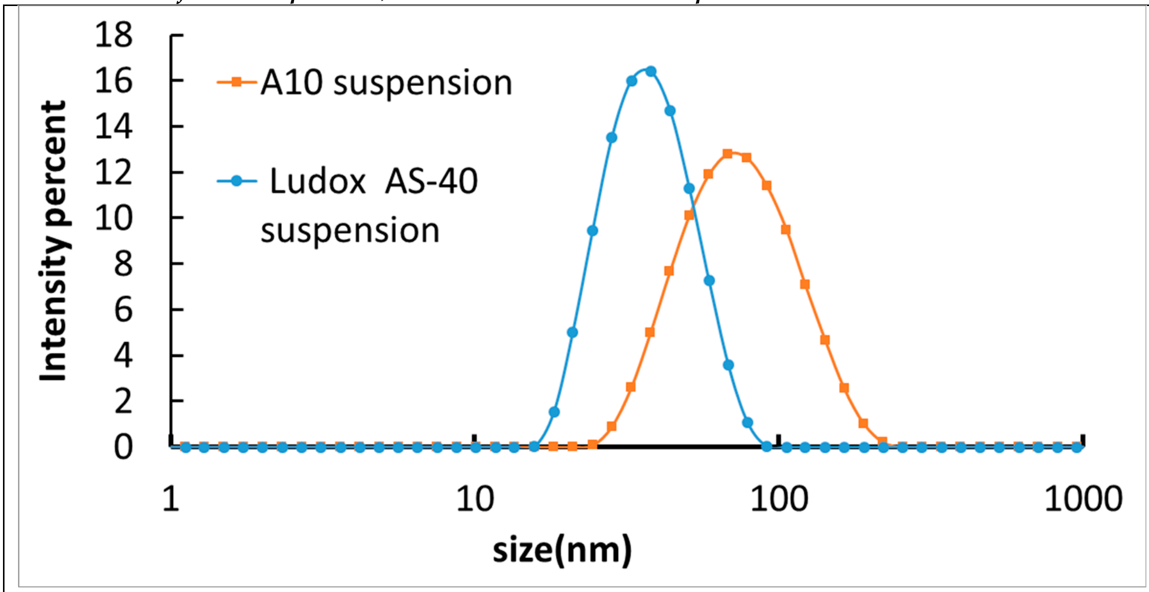

Figure S2: DLS data of NPs suspension in ethanol and in A10 suspension
In ethanol suspension, DLS size is 38±4 and 68±4 nm for the NP and A10 suspensions respectively.

SI3: Histograms of the thickness, RMS roughness and water contact angle evolution after UV irradiation, protocol A and protocol B.

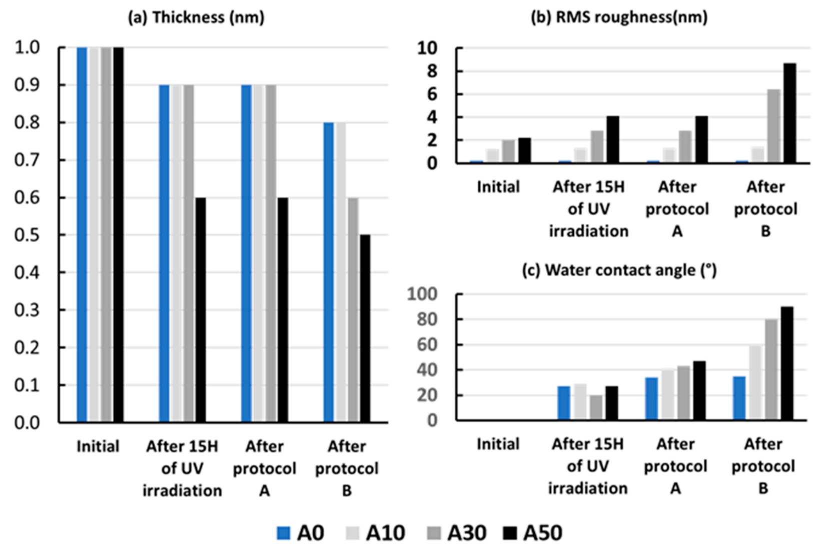

Figure S3: Histograms of the thickness, RMS roughness and water contact angle evolution after UV irradiation, protocol A and protocol B.
Protocol A: UV irradiation + HMDS exposure
Protocol B: UV irradiation+ O_3 exposure + HMDS exposure

SI4: scheme of the evolution of the thickness and roughness after protocol A and protocol B.

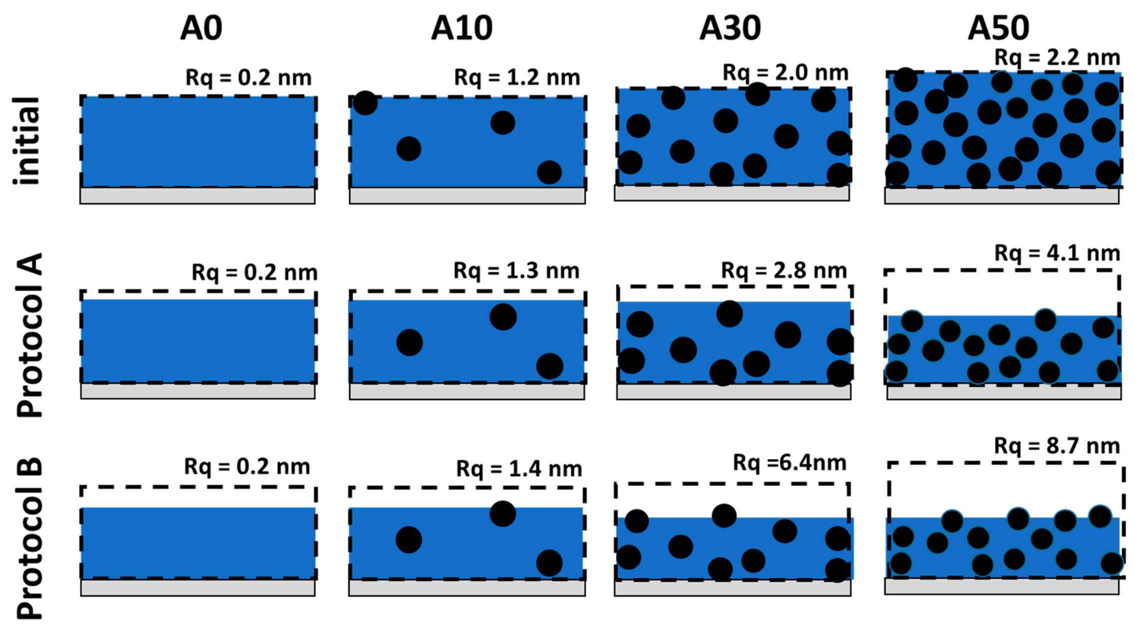

Figure S4: Scheme of the evolution of the thickness and roughness after protocol A and protocol B.
Protocol A: UV irradiation + HMDS exposure;
Protocol B: UV irradiation+ O_3 exposure + HMDS exposure.
The evolution the thickness in terms of film thickness and roughness was not presented for 15 H UV irradiation since the thicknesses and the roughnesses are the same as for protocol A.
